# Parecoxib Shortens the Duration of Acute Postoperative Pain After Laparoscopic-Assisted Vaginal Hysterectomy

**DOI:** 10.3389/fphar.2019.00689

**Published:** 2019-06-18

**Authors:** Shuyi Yang, Wei Xiao, Shijun Wang, Lingzhong Meng, Liane Zhou, Anxia Wan, Yang Liu, Shuai Feng, Tianlong Wang

**Affiliations:** ^1^Department of Anesthesiology, Xuanwu Hospital, Capital Medical University, Beijing, China; ^2^Department of Gynecology and Obstetrics, Xuanwu Hospital, Capital Medical University, Beijing, China; ^3^Department of Anesthesiology, Yale University School of Medicine, New Haven, CT, United States; ^4^National Clinical Research Center for Geriatric Diseases, Xuanwu Hospital, Capital Medical University, Beijing, China

**Keywords:** laparoscopic-assisted vaginal hysterectomy, acute postoperative pain, duration of pain, parecoxib sodium, methylprednisolone

## Abstract

The effect of parecoxib sodium on the duration and severity of acute postoperative pain after laparoscopic-assisted vaginal hysterectomy has been inadequately studied. This randomized, controlled trial compared the effects of parecoxib, methylprednisolone, and placebo on the duration of acute postoperative pain after elective laparoscopic-assisted vaginal hysterectomy. Ninety-four eligible patients were randomized to three groups [parecoxib sodium 40 mg (Group P), methylprednisolone 1 mg/kg (Group M), and saline (Group S)]. The duration of pain during coughing [median (interquartile range)] was significantly lower in Group P than in Group M or Group S [26.0 (5.8–48.0) vs. 48.0 (30.0–55.5) vs. 48.0 (36.0–58.5) h; *p* = 0.025]. The duration of pain during rest was also significantly lower in Group P than in Group M or Group S [5.5 (3.8–21.0) vs. 24.0 (6.0–28.0) vs. 22.0 (5.8–36.0) h; *p* = 0.009]. Compared with those in Group M and Group S, the patients in Group P reported less intense visceral pain during coughing at 12 (*p* = 0.050) and 24 h (*p* = 0.009) as well as at rest at 12 h (*p* = 0.008). Compared with those in Group P and Group S, the patients in Group M showed lower serum C-reactive protein levels and higher blood glucose levels after surgery. No differences were noted in nausea, vomiting, length of hospital stay, wound infection, and delayed wound healing among the groups. Thus, parecoxib sodium reduces the duration and intensity of acute postoperative pain after laparoscopic-assisted vaginal hysterectomy.

## Introduction

Postoperative pain remains a great challenge. A survey revealed that 30% of 5,703 ambulatory patients experienced moderate-to-severe postoperative pain and that laparoscopic procedures were some of the most common reasons underlying this pain (McGrath et al., 2004). In addition to causing unnecessary suffering and discomfort, poorly controlled postoperative pain may also lead to other morbidities, such as ischemic myocardial events (Rawal, 1998).

Parecoxib, a cyclooxygenase-2 selective inhibitor commonly used in the postoperative period, exerts anti-inflammatory effects by inhibiting prostaglandin synthesis (FitzGerald and Patrono, 2001). Although the effect of parecoxib on pain intensity has been reported (Lloyd et al., 2009), there is a lack of information regarding its effect on the duration of postoperative pain. Considering the contraindications of parecoxib, we were seeking for a substitution of parecoxib in postoperative pain management. Methylprednisolone is a glucocorticoid with anti-inflammatory activities. Although glucocorticoids reportedly inhibit spinal cord neuronal nociceptive afferent input and prevent peripheral and central sensitization (Woolf and Chong, 1993), the effects of methylprednisolone on postoperative pain remain controversial. Several trials have demonstrated its analgesic effects (Lunn et al., 2011; Acham et al., 2013); however, contradictory results have also been reported (Aabakke et al., 2014).

We hypothesized that both parecoxib and methylprednisolone alleviate postoperative inflammatory pain owing to their anti-inflammatory activities. This randomized controlled trial compared the effects of parecoxib, methylprednisolone, and placebo on the duration of acute postoperative pain after laparoscopic-assisted vaginal hysterectomy (LAVH). The study also assessed postoperative pain intensity, analgesic consumption, nausea, vomiting, wound infection, delayed wound healing, presence of fever, gastrointestinal recovery, inflammatory factors, blood glucose levels, and length of hospital stay (LOS).

## Methods

### Study Design

This prospective, randomized controlled trial with patient and outcome assessor blinding was approved by the Internal Review Board of Xuanwu Hospital of Capital Medical University, Beijing, China, and was conducted from September 2016 to September 2017. All experimental procedures used were performed in accordance with the guidelines for Good Clinical Practice and the Declaration of Helsinki. Verbal and written informed consents were obtained from all the included patients. This trial was registered at the Chinese Clinical Trial Registry (ChiCTR-IOR-16009152).

### Patients

Patients scheduled for elective LAVH under general anesthesia were included in this study. The other inclusion criteria were as follows: age 18–65 years (McGrath et al., 2004) and American Society of Anesthesiologists (ASA) physical status classification of I to II (Rawal, 1998). The exclusion criteria were as follows: participation in other trials (McGrath et al., 2004), cancer (Rawal, 1998), infection (FitzGerald and Patrono, 2001), smoking history (FitzGerald and Patrono, 2001), body mass index of >35 kg/m^2^ (Lloyd et al., 2009), alcohol or drug use (Woolf and Chong, 1993), chronic pain (Lunn et al., 2011), contraindication for study medication use (Acham et al., 2013), and refusal to participate (Aabakke et al., 2014).

### Randomization and Blinding

The included patients were randomly assigned to three groups (Groups P, M, and S) in a 1:1:1 ratio. Randomization was performed *via* an online randomization software (https://tools.medsci.cn/rand) prepared by an investigator with no clinical involvement in this study. After randomization, based on the randomization list, the study medication was pre-packed by the pharmacy in consecutively numbered boxes. The patients received the treatment corresponding to their group. Information regarding the treatment was concealed in consecutively numbered, sealed, opaque envelopes to enable un-blinding in case of acute complications. The patients and research personnel responsible for outcome assessment were blinded to the groups. The randomization code was broken only after patient enrollment and follow-up had ended.

### Interventions

The anesthesia management protocol was standardized among the groups. Heart rate, non-invasive blood pressure level, pulse oxygen saturation level, nasopharyngeal temperature, end-tidal carbon dioxide level, and bispectral index (BIS) were routinely monitored. Anesthesia was induced using propofol (1.5–2.5 mg/kg), remifentanil (1.5 µg/kg), and rocuronium (0.6 mg/kg). All patients were intubated with an endotracheal tube and ventilated with 40% oxygen in an oxygen–air mixture. Anesthesia was maintained with propofol (4–6 mg/kg/h) and remifentanil (0.2–0.4 µg/kg/min). The propofol infusion was titrated to maintain the BIS value between 40 and 60.

Group P received 40 mg parecoxib sodium (Dynastat, Pfizer, Kalamazoo, USA) in 1 ml of normal saline solution (0.9%) 30 min prior to the end of surgery. Group M received methylprednisolone (Solu-Medrol, Pfizer, Puurs, Belgium) at 1 mg/kg before induction and 1 ml of normal saline solution (0.9%) 30 min prior to the end of surgery. Group S received 1 ml of normal saline solution (0.9%) before induction as well as 30 min prior to the end of surgery. All groups received 0.1 mg/kg of oxycodone (Oxynorm, Mundipharma, Nottinghamshire, UK) and 4 mg of ondansetron 30 min prior to the end of surgery.

Postoperative pain was assessed using the numeric rating scale (NRS; score range 0–10; 0, no pain; 10, worst imaginable pain). Intravenous oxycodone (0.05 mg/kg) was administered if the pain exceeded an NRS score of 3. Postoperative nausea or vomiting was treated with intravenous ondansetron (4 mg). If ondansetron was ineffective, metoclopramide (10 mg) was administered. Patients administered other analgesics, antiemetics, or sedatives during the first 48 h postoperatively were excluded from the study.

### Blood Samples

Venous blood samples were collected before induction (T0, baseline), at the end of surgery (T1), as well as 24 (T2) and 48 h (T3) after surgery. The samples were used for analyzing serum C-reactive protein (CRP) and tumor necrosis factor-α (TNF-α) levels. The samples were centrifuged for 15 min at 3,000 rpm, and the supernatants were frozen at −80°C until laboratory analysis was performed.

### Data Collection

Patient demographic data, including age, body mass index, ASA physical status, and preoperative diagnosis, were recorded 1 day prior to the scheduled surgery. The patients were preoperatively trained by research personnel regarding the use of the NRS. Pain and adverse effects, such as nausea, vomiting, wound infection, delayed wound healing, and fever, were evaluated at the end of surgery and at 1, 2, 3, 4, 6, 12, 24, and 48 h postoperatively. Pain was assessed using the NRS during coughing (patients were told to cough) and at rest, as described previously (Luscombe et al., 2010; Hwang et al., 2014; Kleif et al., 2017). The duration of acute postoperative pain (from the end of surgery to the time point when the NRS score was 0) during coughing and at rest as well as the location of pain (incisions, abdomen, or no pain) were recorded. If the NRS score was >0 at discharge, the duration of pain was calculated from the end of surgery to discharge. To reduce the risk of adverse outcomes resulting from the insufficient postoperative pain control and enhance the quality of patients’ life, our target was to reduce or eliminate postoperative pain before discharge (Apfelbaum et al., 2012). Considering the potential glucose-increasing effect of methylprednisolone, blood glucose levels were measured before induction (T0), at the end of surgery (T1), and at 1 h after surgery using a glucometer (Accu-Chek Performa, Accu-Chek Inform II test strips; Roche Diagnostics, Indianapolis, IN, USA). The serum CRP and TNF-α levels were determined using an enzyme-linked immunosorbent assay (Biokits Tech, Inc., Beijing, China), which was performed according to the manufacturer’s instructions. All samples were analyzed at a dilution resulting in levels within the standard curve range.

The primary outcome was the duration of pain (during coughing or at rest) after surgery. The secondary outcomes were pain intensity during coughing or at rest 0–48 h postoperatively (NRS), oxycodone consumption, adverse effects (nausea, vomiting, wound infection, delayed wound healing, and fever), gastrointestinal recovery, serum CRP and TNF-α levels, blood glucose levels, and LOS.

### Statistical Analysis

The study was powered for the primary outcome. According to a pilot study, a sample size of 29 patients in each group would be required (90% power; alpha error of 0.05; two-sided test) to detect a difference in the duration of pain during coughing [Group S, 31.5 (5.7) h vs. Group P, 7.5 (8.4) h vs. Group M, 27.0 (15.4)]. Assuming a 20% dropout rate, we planned to recruit a total of 105 patients.

Data are presented as mean and SD, median and interquartile range (IQR), or frequency, as appropriate. Continuous outcome variables were tested for normality of distribution *via* visual inspection and the Kolmogorov–Smirnov test. Data that followed a normal distribution (demographic and surgical data, serum CRP and TNF-α levels, flatus time, LOS, and blood glucose levels) were analyzed using analysis of variance (ANOVA). Relationships between the duration of pain and inflammatory factors were analyzed using Spearman’s rank correlation coefficient. Data that followed a non-normal distribution (duration of pain after surgery, NRS pain score, and oxycodone consumption) were analyzed using the Kruskal–Wallis test. If statistical significance was observed in the analyses among the three groups, the differences in data between the groups were further assessed using the Student–Newman–Keuls test or Mann–Whitney *U*-test. Categorical data (ASA, incidence of adverse effects, and complications) were analyzed using the chi-square test. All statistical analyses were performed using SPSS version 19.0 for Windows (IBM Corp., Armonk, NY, USA). A *p*-value of <0.05 was considered statistically significant.

## Results

**Figure 1:**
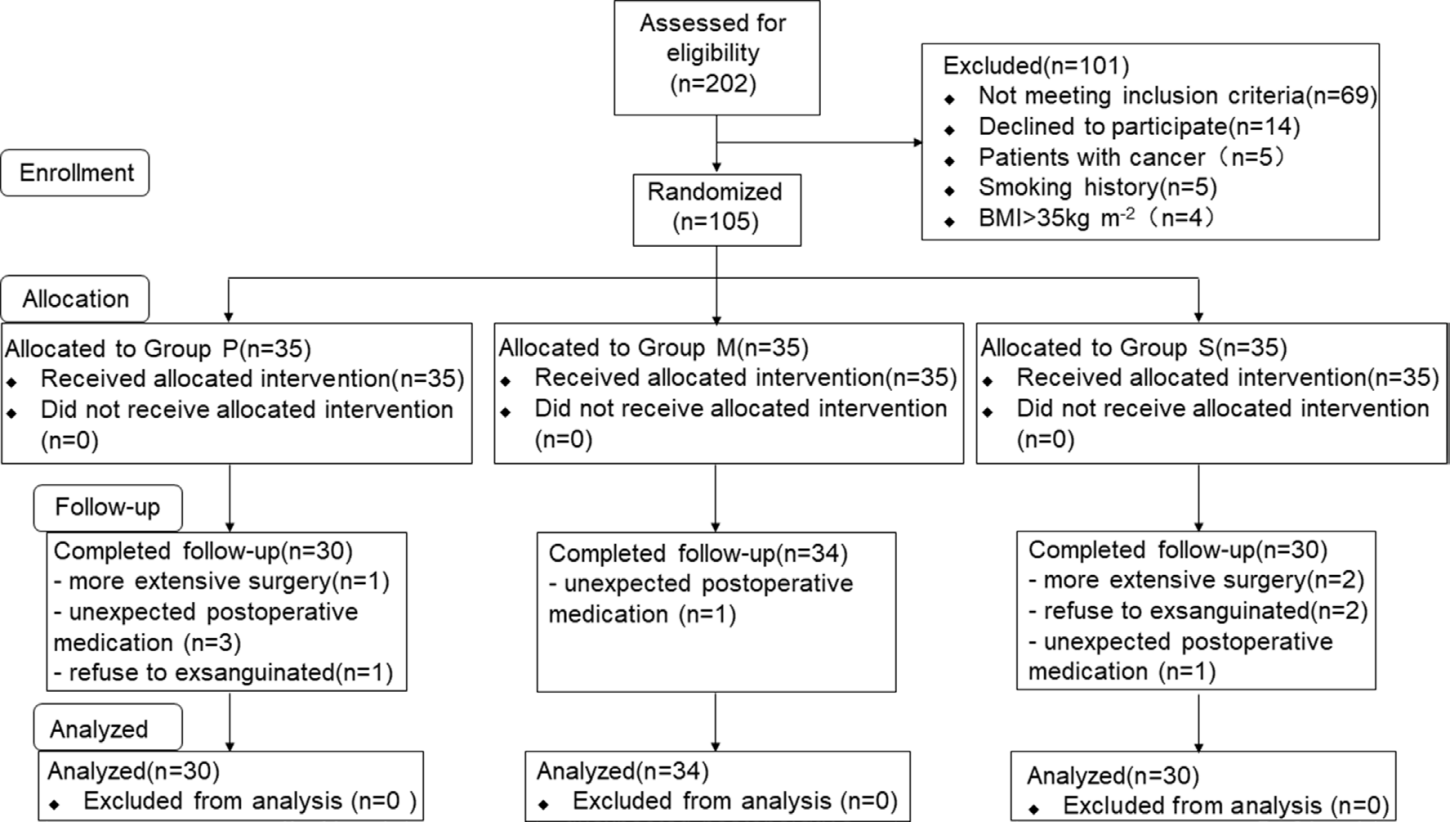
Study flow diagram. BMI, body mass index.

A total of 202 consecutive patients scheduled for LAVH were considered for inclusion, and eventually, 105 patients were included in this study and randomly assigned to three groups (**Figure 1**). Of the 105 patients, 11 were excluded from the analysis (three for extensive surgery, three for refusing to be exsanguinated, and five for unexpected postoperative medications), and consequently, 94 patients were finally included. Of the 94 patients, 30, 34, and 30 were included in Groups P, M, and S, respectively. Patient characteristics are summarized in **Table 1**. No significant differences were observed in the demographic and surgical data among the study groups.

**Table 1:** Patients’ demographic characteristic and surgical data.

Characteristics	Group P	Group M	Group S	*p* value
Age, y	52.5 (7.3)	51.2 (6.3)	50.2 (6.5)	0.403
ASA group, I/II	20/10	23/11	19/11	0.931
BMI, kg/m^2^	24.2 (2.7)	25.3 (3.3)	26.1 (3.1)	0.055
Duration of operation, min	78.1 (25.5)	72.8 (20.1)	79.1 (28.7)	0.548
Total remifentanil consumption, μg	1,623.7 (450.4)	1,621.9 (407.0)	1,740.7 (901.0)	0.692

Data are mean (SD) or frequencies. BMI, body mass index; ASA, American Society of Anesthesiologists physical status.

The duration of pain of the different groups following surgery is presented in **Table 2**. The duration of pain during coughing after surgery was significantly lower in Group P [26.0 (5.8–48.0) h] than in Group M [48.0 (30.0–55.5) h; *p* = 0.028] and Group S [48.0 (36.0–58.5) h; *p* = 0.013]; the durations were similar between Groups M and S (*p* = 0.714). The duration of pain during rest was significantly lower in Group P than in Group M (*p* = 0.008) and Group S (*p* = 0.008); the durations were similar between Groups M and S (*p* = 0.939).

**Table 2:** Duration of pain during coughing and at rest.

Duration of pain	Group P	Group M	Group S	*p* value
During coughing, h	26.0 (5.8–48.0)	48.0 (30.0–55.5)	48.0 (36.0–58.5)	0.025
At rest, h	5.5 (3.8–21.0)	24.0 (6.0–28.0)	22.0 (5.8–36.0)	0.009

Data are median and IQR (interquartile range).

**Figure 2:**
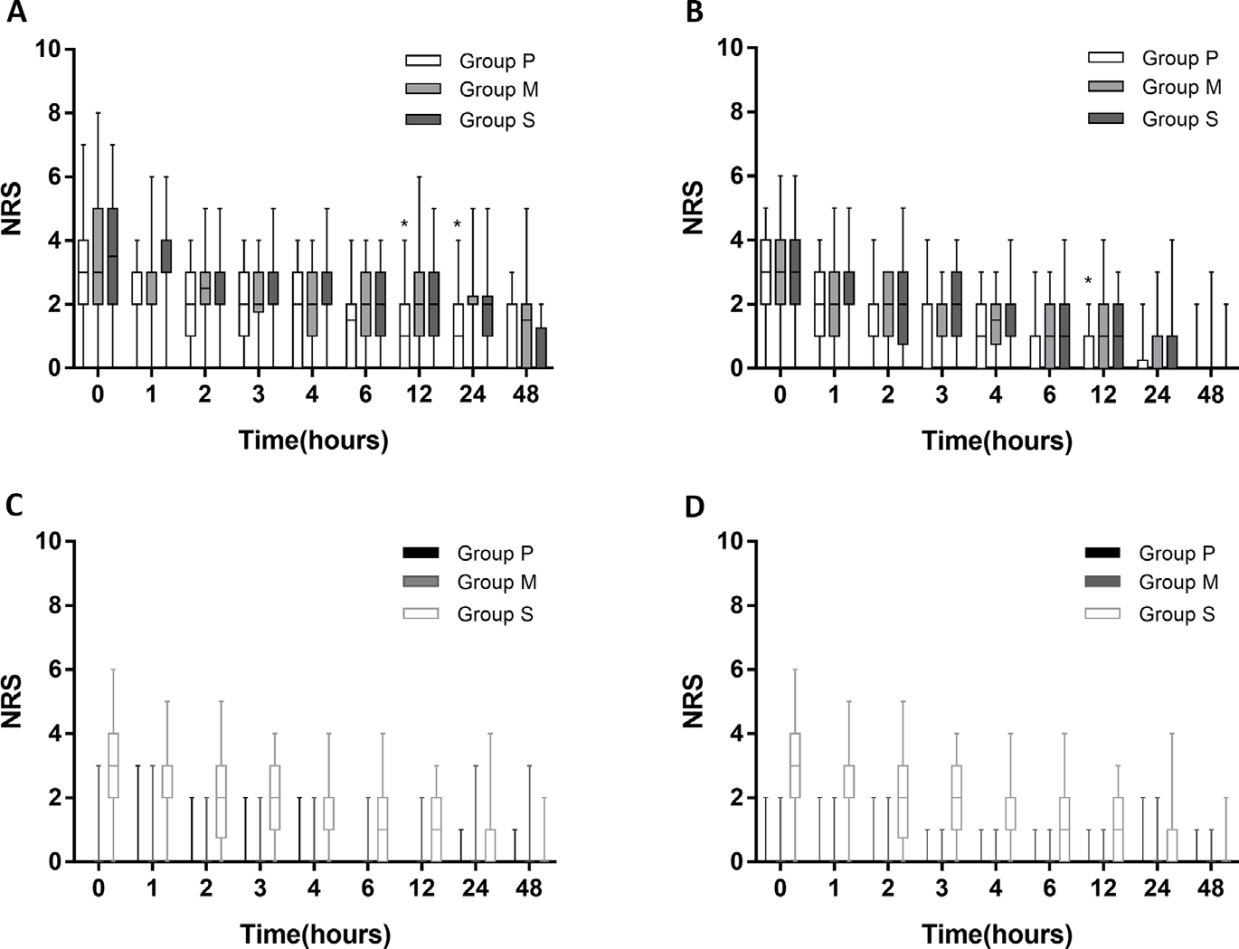
NRS score following surgery. **(A)** NRS score of visceral pain intensity during coughing in 3 groups: parecoxib (Group P), methylprednisolone (Group M), and placebo (Group S). **(B)** NRS score of visceral pain intensity at rest. **(C)** incisional pain during coughing. **(D)** incisional pain at rest. Asterisks represented statistically significant difference between the groups: *p < 0.05.

Pain intensities following surgery in the three groups are shown in **Figure 2**. Pain intensities (NRS scores) were measured at 0, 1, 2, 3, 4, 6, 12, 24, and 48 h after surgery. At 12 h after surgery, visceral pain during coughing was significantly lower in Group P than in Group S (*p* = 0.018). At 24 h after surgery, visceral pain during coughing was significantly lower in Group P than in Group M (*p* = 0.004) and Group S (*p* = 0.014) (**Figure 2A**). At 12 h after surgery, visceral pain at rest was significantly lower in Group P than in Group M (*p* = 0.010) and Group S (*p* = 0.005) (**Figure 2B**). The NRS scores of incisional pain during coughing and at rest were similar among all the groups (**Figure 2C and D**).

The secondary outcomes are presented in **Table 3**. Postoperative oxycodone consumption was similar among the three groups (*p* = 0.099). There were no significant differences in the baseline serum CRP and TNF-α levels among the three groups before surgery. Repeated-measures ANOVA for CRP levels revealed a significant main effect of treatment (*F* = 125.8; *p* < 0.001), a significant effect of time (*F* = 69.2, *p* < 0.001), and a time-by-treatment interaction (*F* = 3.4; *p* = 0.004). At 24 and 48 h after surgery, the serum CRP level was significantly lower in Group M than in Groups P or S (24 h, *p* < 0.001; 48 h, *p* = 0.005). The main effect of treatment on the TNF-α levels yielded a significant *F* ratio (*F* = 97.6; *p* < 0.001) and a significant effect of time (*F* = 39.0; *p* < 0.001); however, the time-by-treatment interaction was not significant (*F* = 0.04; *p* = 0.715). Direct comparisons indicated that compared with the preoperative levels in all groups, the TNF-α levels significantly increased at 24 and 48 h after surgery. The TNF-α levels among the three groups at each time point demonstrated no significant differences. No correlations were observed between the CRP or TNF-α level and the duration of pain during coughing (CRP: *p*_T1_ = 0.498, *p*_T2_ = 0.751, *p*_T3_ = 0.856; TNF-α: *p*_T1_ = 0.306, *p*_T2_ = 0.317, *p*_T3_ = 0.244) and at rest (CRP: *p*_T1_ = 0.350, *p*_T2_ = 0.251, *p*_T3_ = 0.770; TNF-α: *p*_T1_ = 0.966, *p*_T2_ = 0.219, *p*_T3_ = 0.106).

**Table 3:** Secondary outcomes of study.

	Group P	Group M	Group S	*p* value
Oxycodone consumption, mg	2.3 (3)	2.0 (6.5)	4.8 (10)	0.099
Serum CRP level				
CRP, T0, ng/mL	615.6 (724.3)	520.8 (513.1)	546.8 (642.6)	0.827
CRP, T1, ng/mL	622.5 (731.3)	483.9 (444.9)	536.5 (606.7)	0.652
CRP, T2, ng/mL	5,859.7 (3387.5)*	2,822.0 (2361.3)*	5,408.7 (3456.3)*	0.000
CRP, T3, ng/mL	8,699.3 (6283.6)*	4,893.7 (4118.4)*	9,375.7 (6915.8)*	0.005
Serum TNF-α level				
TNF-α, T0, pg/mL	18.9 (18.6)	17.3 (16.7)	16.2 (5.8)	0.771
TNF-α, T1, pg/mL	18.6 (3.6)	16.3 (15.5)	16.3 (7.8)	0.791
TNF-α, T2, pg/mL	40.5 (29.0)*	32.9 (30.7)*	33.1 (16.6)*	0.443
TNF-α, T3, pg/mL	52.1 (44.3)*	43.1 (38.1)*	41.8 (22.7)*	0.485
PONV, *n* (%)	12 (40)	10 (29)	14 (47)	0.357
Flatus time, h	24.2 (5.7)	26.1 (6.7)	24.9 (5.4)	0.450
LOS, days	4.5 (0.9)	4.5 (1.0)	4.8 (1.3)	0.317
BG, T0, mmol/L	5.4 (0.8)	5.4 (0.9)	5.7 (1.3)	0.529
BG, T1, mmol/L	5.4 (0.8)	5.9 (1.1)	5.4 (1.1)	0.042
BG, 1 h after the end of operation, mmol/L	5.9 (1.1)	6.9 (1.5)	6.0 (1.3)	0.001

Data are mean (SD) or median and IQR (interquartile range) or frequencies. *Compared with T0, p < 0.05. CRP, C-reactive protein; TNF-α, tumor necrosis factor alpha; LOS, length of hospital stay; BG, blood glucose; PONV, postoperative nausea and vomiting.

Postoperative nausea and vomiting (PONV) occurred in 12 patients from Group P, 10 from Group M, and 14 from Group S. The incidence of PONV was similar among the three groups (*p* = 0.357). The flatus time and LOS were similar among the three groups (*p* = 0.450 and *p* = 0.317, respectively). The blood glucose level before induction (T0) was also similar among the three groups (*p* = 0.529). However, the blood glucose levels at the end of surgery and at 1 h after surgery were significantly higher in Group M than in Groups P or S. No patient from Group P, one patient from Group M, and three patients from Group S had fever (axillary temperature > 38°C) or leukocytosis (leucocyte count > 10 × 10^9^/L) after surgery. No patient had wound infection or delayed wound healing.

## Discussion

The present trial demonstrated that intraoperative administration of parecoxib sodium 40 mg (Group P) significantly shortened the duration of pain during coughing and at rest after LAVH and that it reduced the intensity of acute visceral but not incisional pain. In Group P, the duration of pain during coughing and at rest was 26 and 5.5 h, respectively, and the mean LOS after surgery was 2.6 days, indicating that Group P achieved the clinical target of “eliminate postoperative pain before discharge” (Apfelbaum et al., 2012).

Few trials have mentioned the impact of parecoxib on the duration of acute postoperative pain; the mechanism associated with the effect of parecoxib on the pain process remains unclear. The analgesic effect of parecoxib is attributable to the blockage of the arachidonic acid cascade and production of prostaglandins (FitzGerald and Patrono, 2001), the reductions in both basal and enhanced prostaglandin release in the spinal cord (Vanegas and Schaible, 2001), and the further prevention of peripheral sensitization (Woolf and Chong, 1993). Prostaglandins, which are metabolites of arachidonic acid, play important roles in modulating inflammatory and nociceptive processes (Vane et al., 1998), directly exciting nociceptors, and potentiating the sensitizing effects of other pain mediators (Vanegas and Schaible, 2001; Moriyama et al., 2005; Wang et al., 2007). A study concerning the mechanism of PGE2-prolonged nociceptor sensitization reported that synthesis stimulation and anterograde axonal trafficking to increase EP4 availability may be one explanation (St-Jacques and Ma, 2014). Considering our result that the duration of pain was shortened with parecoxib, we suspect that the inhibition of PGE2-prolonged sensitization of the nociceptive dorsal root ganglion by parecoxib will help reduce pain processing.

The potential side effects should be considered when choosing analgesics. Because parecoxib is contraindicated in patients with ischemic heart disease, cerebrovascular disease, peripheral vascular diseases, congestive heart failure, history of sensitivity to sulfonamides, and bypass operation of the coronary artery (Lloyd et al., 2009), a substitute drug is needed. It still remains unclear whether glucocorticoids can be used as substitutes for parecoxib (Lunn et al., 2011; Aabakke et al., 2014). Thus, in this study, we used methylprednisolone and compared its effects with those of parecoxib and placebo.

The analgesic effects of glucocorticoids after laparoscopy surgery are controversial. One study demonstrated that dexamethasone at 0.1 mg/kg reduced pain at 2, 6, and 12 h after surgery (Mohtadi et al., 2014). Another study found no differences between placebo and dexamethasone at different doses (5, 10, and 15 mg) (Jokela et al., 2009). A meta-analysis involving 5,796 patients from 45 studies concluded that those who received dexamethasone (1.25–20 mg) had lower pain scores at 2 and 24 h after surgery, a longer time to the first dose of analgesic, and reduced opioid consumption with no differences among doses (Waldron et al., 2013). Because methylprednisolone has a more rapid onset time than has dexamethasone and a perioperative single dose of methylprednisolone does not increase the occurrence of adverse effects (Sauerland et al., 2000), we used methylprednisolone instead of dexamethasone in this study.

Data concerning the analgesic effects of methylprednisolone are sparse and inconsistent (Lunn et al., 2011; Acham et al., 2013; Aabakke et al., 2014). Methylprednisolone has shown analgesic effects in orthopedic and dental surgeries. Rytter et al. claimed that administration of 125 mg of systemic methylprednisolone could reduce postsurgical pain and decrease opioid requirement after knee arthroplasty (Rytter et al., 2017). This pain-reducing effect has also been reported in third molar surgery (Acham et al., 2013). In contrast, Aabakke et al. (2014) randomized 59 patients undergoing abdominal hysterectomy to receive 125 mg of methylprednisolone or saline and assessed postoperative pain in the first 24 h at rest and during mobilization using a visual analog scale. Aabakke et al. found that methylprednisolone had no effect on postoperative pain after surgery, and this finding corroborates with our results. The pain-reducing effects of methylprednisolone in orthopedic and dental surgeries and not gynecologic surgery may be associated with its anti-inflammatory effects that reduce local swelling and edema (Holte and Kehlet, 2002; Lunn et al., 2011).

Some studies have suggested the opioid-sparing effects of parecoxib (Ng et al., 2003; Parsons et al., 2016) and glucocorticoids (Jokela et al., 2009; De Oliveira et al., 2011; Mohtadi et al., 2014). However, our study demonstrated a tendency of lower oxycodone consumption in both the parecoxib and methylprednisolone groups compared with the saline group (median, 2.3 vs. 2.0 vs. 4.8 mg), but no statistical difference was noted between the groups. Our sample size was calculated according to the primary outcome (duration of postoperative pain) and was not powered to demonstrate a difference in the oxycodone consumption. A larger sample size may help identify a difference in oxycodone consumption.

Surgical trauma induces an inflammatory state that is characterized by the local release of inflammatory proteins and cytokines, which enter the circulation and systemically spread (Bautmans et al., 2010). TNF-α is a major mediator that responds to tissue damage secondary to surgery (Hackam and Rotstein, 1998). CRP is an acute phase reactant, and its level increases in response to inflammatory stimuli (Rosalki, 2001). Inflammation may enhance postoperative pain (Gaskell et al., 2017); however, in the present study, we found no correlations between the levels of postoperative inflammatory cytokines (TNF-α and CRP) and the duration of pain. Our findings suggest that the pain process is irrelevant with respect to these two systemic inflammatory indicators. Considering that parecoxib shows antinociceptive and anti-inflammatory effects by inhibiting prostaglandin synthesis (Ricciotti and FitzGerald, 2011), prostaglandins could be highly sensitive inflammatory mediators and major enhancers of nociceptive responses to surgery (Brown et al., 2018). Further studies are required to confirm the relationships among prostaglandins, surgery, and pain.

Elevated blood glucose levels and immunosuppression are potential concerns for glucocorticoid use. A previous meta-analysis investigated these risks and concluded that perioperative administration of glucocorticoids did not affect wound infection but caused a clinically insignificant increase in the peak glucose level in noncardiac surgery (Toner et al., 2017). There is no conclusive evidence that suggests that a single glucocorticoid dose is associated with hyperglycemia, and this may contribute to postoperative morbidity (Sauerland et al., 2000). In the present study, 1 mg/kg of methylprednisolone increased the postoperative glucose level when compared with the level in the control group; however, this did not seem to be clinically significant. In addition, methylprednisolone did not increase the risk of fever or delayed wound healing, which is similar to the findings of previous studies (Sauerland et al., 2000).

The present study was implemented in patients who underwent LAVH and demonstrated findings similar to those of a previous study (Barton et al., 2002), which also reported on a laparoscopy procedure; hence, the present study suggests that other patients scheduled for laparoscopy procedure also benefit from parecoxib administration and additional oxycodone for treating visceral pain.

The present study had some limitations, such as the lack of power to demonstrate differences in the secondary outcomes. Furthermore, for optimal comparison, it would have been ideal to maintain the same administration timings for parecoxib and methylprednisolone.

## Conclusion

Intraoperative parecoxib administration significantly shortened the duration of pain and reduced the intensity of patient-reported pain after LAVH. This effect cannot be duplicated following the administration of methylprednisolone, although methylprednisolone significantly lowered the postoperative CRP levels. The CRP and TNF-α levels and pain durations are not correlated. Further research on the correlation between systemic inflammation and the pain process is warranted.

## Data Availability Statement

All datasets generated for this study are included in the manuscript and the supplementary files.

## Ethics Statement

This study was carried out in accordance with the recommendations of Internal Review Board at Xuanwu Hospital of Capital Medical University with written informed consent from all subjects. All subjects gave written informed consent in accordance with the Declaration of Helsinki. The protocol was approved by the Internal Review Board at Xuanwu Hospital of Capital Medical University.

## Author Contributions 

SY: Design and initiation of the study, patient recruitment, monitoring of processes, compilation of the CRF database, statistical analyses, composition of the first draft of the manuscript, and preparation of the figures. WX: Design and initiation of the study, patient recruitment, clinical support and oversight, monitoring of processes, statistical analyses, composition of the first draft of the manuscript, and preparation of the figures. SW: Clinical support and oversight. LM: Statistical analyses and preparation of the figures. LZ: Clinical support and oversight. AW: Clinical support and oversight. YL: Statistical analyses. SF: Initiation of the study and statistical analyses. TW: Study concept, design, and initiation of the study. Revision and approval the final version of the paper: all authors.

## Funding

Funding for research was provided by the Beijing Municipal Administration of Hospitals Clinical Medicine Development of Special Funding Support (ZYLX201818, ZYLX201706), National Clinical Research Center for Geriatric Disorders, Beijing, China, acknowledgements-Beijing Municipal commission of Health and Family Planning (PXM2017_026283_000002), Beijing Yang Core Scholar Research Grant (2014000020124G160), Beijing 215 High Level Healthcare Talent Plan Academic Leader (No. 008-0027), and Beijing Municipal Administration of Hospital Ascent Plan (DFL20150802).

## Conflict of Interest Statement

The authors declare that the research was conducted in the absence of any commercial or financial relationships that could be construed as a potential conflict of interest.
